# The Royal College of Surgeons of England

**Published:** 1900-01-27

**Authors:** 


### The Hospital

A Journal of

The Medical Sciences and Hospital Administration.

Vol. XXVII.—No. 696.] Edited by Sir Henry Burdett, K.C.B., and Solomon C. Smith, M.D., M.R.C.P. [January 27, 1900.

### THE ROYAL COLLEGE OF SURGEONS OF ENGLAND.

At the quarterly meeting of the Council of the Royal College of Surgeons of England, held on the 18th instant, important resolutions were passed with regard to the conduct of Fellows or Members of the College who were associated with any medical aid societies or institutions which systematically canvass and advertise for the purpose of procuring patients, and also with regard to the conduct of certain dentists who, possessing no qualification from any licensing body, but being registered as having been in practice prior to the Act of 1878, nevertheless adopt titles which cause it to be inferred that they are connected with one of the Colleges of Surgeons of the United Kingdom, and who thus infringe the privileges of the licentiates in dental surgery of these colleges.

The former of these questions was brought before the Council in connexion with a resolution, carried at the annual meeting of Fellows and Members of the College, which set forth that no Fellow or Member "ought to be allowed" to act as medical officer to "any association which has for one of its objects the provision of medical attendance, and which, in pursuance of that object, advertises and canvasses for patients," and by which the Council of the College was "once more urged" to announce that the holding of such appointments is "contrary to the declaration made by Fellows and Members on their admission." This resolution had been considered by the Discipline Committee of the Council, who recommended the Council to express their "formal disapproval" of the conduct objected to, and this recommendation was adopted. So definite an expression of opinion, on the part of the rulers of a leading medical corporation, will unquestionably carry considerable weight, and will tend steadily to discourage the acceptance of office under the societies and conditions which are condemned; but it will be observed that the Council, no doubt acting under legal advice, did not see its way to go the whole length of the original resolution. The "declaration" pledges the new member of the College to "demean himself honourably in the practice of his profession, and to the utmost of his power to maintain the dignity and welfare of the College." The power of the Council to take action against any member under the by-laws only arises in the event of his having been judged by the Council, after due inquiry, to have been guilty of "disgraceful conduct in any professional respect." Assuming that a member of the College was salaried surgeon to a medical aid society, with no duties but those of attending to the sick, and that the managers of the society sought to increase the membership by canvassing, an act in which the surgeon himself took no part, it is extremely doubtful whether a court of law would uphold the Council in deciding that it was "disgraceful in a professional respect" for the member to hold office under a committee or a management of whose conduct, in a matter outside of his own province or his professional work, he himself might entirely disapprove.

In the case of the registered but unqualified dentists, the legal position seems more secure, and the Council of the College has called the attention of the General Medical Council to the facts, and has urged the latter body to declare the improper assumption of titles to be disgraceful within the meaning of the Dentists Act, and to afford ground for the erasure of the names of delinquents from the Register. It appears that the persons implicated have adopted such titles or descriptions as "R.D.S., R.C.S.Eng.," or "R.D.S.Eng.," or "R.D.S.E.," or "Registered by the Royal College of Surgeons," when in point of fact they have no connection with any licensing body whatever. It is well known, moreover, that the interpretation placed upon the words "in practice prior to the passing of this Act," was extremely lax; that they were held to apply to every person who could show that he had received payment for the extraction of a tooth before the appointed time, that for a certain period they covered even those who on the specified date had commenced an apprenticeship to any one who called himself a dentist, and that, as a general result, they placed upon the Dental Register a large number of persons who were utterly unfit to discharge professional duties or to be entrusted with professional responsibilities. There can be no reasonable doubt that the use of the letters or titles referred to is a false pretence, intended and calculated to deceive the public; nor can there be any doubt at all that the use of false pretences is "disgraceful" in a professional man. The Dentists Act gives to the General Medical Council powers more extensive than those which it possesses under the Medical Acts, for while, under the latter, erasure from the Register can only be ordered on proof of "infamous conduct in any professional respect," it is sufficient, under the former, for the conduct to be "infamous or disgraceful." Neither word lends itself easily to definition, but it is certain that the latter would cover misconduct, which could hardly be said to satisfy the meaning of the former.

